# Syphilis vaccine: challenges, controversies and opportunities

**DOI:** 10.3389/fimmu.2023.1126170

**Published:** 2023-04-06

**Authors:** Carlos Ávila-Nieto, Núria Pedreño-López, Oriol Mitjà, Bonaventura Clotet, Julià Blanco, Jorge Carrillo

**Affiliations:** ^1^ IrsiCaixa AIDS Research Institute, Badalona, Spain; ^2^ Autonomous University of Barcelona, Cerdanyola del Vallès, Catalonia, Spain; ^3^ Skin Neglected Tropical Diseases and Sexually Transmitted Infections Department, Germans Trias i Pujol Hospital, Badalona, Spain; ^4^ Fight Infections Foundation, Germans Trias i Pujol Hospital, Badalona, Catalonia, Spain; ^5^ Centre for Health and Social Care Research (CESS), Faculty of Medicine, University of Vic – Central University of Catalonia (UVic – UCC), Vic, Catalonia, Spain; ^6^ Germans Trias i Pujol Research Institute (IGTP), Badalona, Spain; ^7^ CIBERINFEC, Instituto de Salut Carlos III (ISCIII), Madrid, Spain

**Keywords:** syphilis, treponema pallidum, vaccine, immune response, outer membrane proteins

## Abstract

Syphilis is a sexually or vertically (mother to fetus) transmitted disease caused by the infection of *Treponema pallidum* subspecie *pallidum* (TPA). The incidence of syphilis has increased over the past years despite the fact that this bacterium is an obligate human pathogen, the infection route is well known, and the disease can be successfully treated with penicillin. As complementary measures to preventive campaigns and early treatment of infected individuals, development of a syphilis vaccine may be crucial for controlling disease spread and/or severity, particularly in countries where the effectiveness of the aforementioned measures is limited. In the last century, several vaccine prototypes have been tested in preclinical studies, mainly in rabbits. While none of them provided protection against infection, some prototypes prevented bacteria from disseminating to distal organs, attenuated lesion development, and accelerated their healing. In spite of these promising results, there is still some controversy regarding the identification of vaccine candidates and the characteristics of a syphilis-protective immune response. In this review, we describe what is known about TPA immune response, and the main mechanisms used by this pathogen to evade it. Moreover, we emphasize the importance of integrating this knowledge, in conjunction with the characterization of outer membrane proteins (OMPs), to expedite the development of a syphilis vaccine that can protect against TPA infection.

## 1 Introduction

Syphilis is a sexual transmitted infection caused by the spirochete *Treponema pallidum* subspecie *pallidum* (TPA). Although the origin of the syphilis epidemic is unclear, the first cases of the disease were reported in Europe in the late 15^th^ century (1, 2). In 1905, Schaudinn and Hoffmann discovered the bacteria causing this venereal disease, and in 1943, the first cases of syphilis were successfully treated with penicillin (3). Over half a century later, penicillin remains one of the most efficacious treatments (4).

Syphilis and the three nonvenereal treponematoses (yaws, bejel and pinta) were at first believed to be caused by the same agent, despite the fact that their clinical manifestations are different. Since pathogenic treponemes are morphologically and antigenically very similar (> 95% DNA homology), genomic sequencing was needed to identify distinct subspecies of *Treponema pallidum* that cause nonvenereal treponematoses: *Treponema pallidum endemicum* (causative agent of bejel), and *Treponema pallidum pertenue* (causative agent yaws). Pinta is caused by a different species of spirochete bacteria, namely *Treponema carateum (* 4, 5). All of them are obligate human pathogens characterized by its invasiveness and immune evasiveness (6–9).

A syphilis infection consists of three main stages (5). Primary syphilis usually begins approximately 2-3 weeks after contact with the pathogen, and it is characterized by the presence of an ulcerated lesion, called chancre. Typically, this lesion appears on the genital area, or other body parts related to sexual contact, usually accompanied by regional lymphadenopathy (5, 10). In the absence of treatment, primary lesion resolves spontaneously in 3-6 weeks. Secondary syphilis develops as a result of bacteria dissemination (10). Clinical manifestations include malaise, headache, fever, diffuse lymphadenopathy, and maculopapular rashes with either discrete or widespread body involvement (5, 10). Unless treated, secondary lesions can take up to several months to resolve. The disease then enters in a latent stage without exhibiting clinical manifestations (5, 6, 10). It remains unclear how TPA establishes latency, and which tissues or organs act as reservoirs (6). A relapse of infection from these reservoirs can occur in 25% of untreated patients within two years after secondary syphilis resolution, and present new secondary-like clinical manifestations (6, 10). After years or even decades, 15-40% of untreated and latently infected individuals will develop tertiary syphilis. This stage involves serious cardiovascular, neurological, bony and visceral affections that may eventually result on death of infected individuals (5, 10). Remarkably, penicillin administration prevents syphilis progression to its secondary and tertiary stages, and cures the infection. However, treated patients remain susceptible to reinfection, because the previous one fails to produce a protective immune response (11–13).

According to WHO data, in 2016, 19.9 million people had syphilis, and there were 6.3 million of new cases per year (14). The prevalence of congenital syphilis was 0.69% in 2016, with a rate of 473 cases per 100.000 live births (15). Syphilis is considered the second cause of stillbirths after malaria (16). A high prevalence of syphilis is observed in low-income countries, and it has been rising in high-middle income states during the last decade (5, 14, 17, 18). Particularly, this increase has been observed in men who have sex with men (MSM) with multiple sexual partners, as well as sexual networks of heterosexual individuals. In this setting, syphilis is also associated with higher risk of HIV infection (5, 17–19).

Importantly, although syphilis can be easily diagnosed, treated with an inexpensive antibiotic, and no animal reservoir has been identified to date, syphilis continues to be a significant global health problem. Therefore, the development of a syphilis vaccine is urgently needed to complement disease control and prevention measures. In this review, we focus on: syphilis vaccine development, use of outer membrane proteins (OMPs) as putative immunogens, anti-TPA immune responses, as well as TPA evasion mechanisms, to provide information about potential antigen selection in future vaccine and immunization strategies.

## 2 *Treponema pallidum* subsp. pallidum

TPA is a flat-wave spirochete of 5-15 μm and 0.2 μm of diameter (20) that belongs to Spirochaetaceae family, particularly *Treponema* genus. Treponemes are usually classified as Gram-negative bacteria due to their double membrane structure (21). However, the composition of their outer membrane (OM) is noticeably different (22, 23). The TPA OM is characterized by the paucity of surface-exposed proteins (24–26), the presence of phosphatidylcholine, phosphatidylglycerol, phosphatidylserine, and the lack of cardiolipins (23) and lipopolysaccharide (LPS) (27, 28). Interestingly, cardiolipins are present in the cytoplasmic membrane (23), and represent the main lipid antigen targeted by anti-TPA antibodies of infected individuals (23). However, other constituents of the OM (e.g. glycolipids) do not present immune reactivity (23, 29). The lipid composition and poor protein content of OM are key features of TPA, and may contribute to the poor immunogenicity of this pathogen (22–24, 26).

TPA is actively motile, although its motile system differs from other flagellar bacteria (30). Axis filaments (known as endoflagella) are found in the periplasmatic space and extend from cell poles through the entire cell body length (31). These filaments comprise three core protein (FlaB1, FlaB2, and FlaB3) and an external protein, which surround the filament core (FlaA) (28, 31). When endoflagella rotate in one direction, the cell body moves in the opposite one. This torsion results in a corkscrew-like motion that, together with the action of metalloprotease and adhesin proteins, allows spirochetes to cross tissues and disseminate through the body (32, 33).

Until recently, TPA had not been grown *in vitro (* 34), and bacteria propagation in rabbits was the only strategy to obtain sufficient live and infective organisms for experimental investigation (35). The lack of the tricarboxylic acid cycle and microaerophilic requirements make this bacterium totally dependent on host cells for the acquisition of purines, pyrimidines, and most amino acids (6, 8, 28). Therefore, TPA cannot survive outside the host, and loses its infectious capability within few hours (8, 36).

Besides humans, rabbits are one of the few mammalians that are susceptible to TPA infection, and has become the reference animal model to study syphilis immune protection. After infection, rabbits develop primary and secondary stage-like clinical signs, and a humoral response similar to the one observed in humans (37–39). Intradermal TPA inoculation induces dermal lesions that resemble human chancres, and bacteria can disseminate to distal organs, mainly secondary lymphoid organs (i.e. spleen) (32, 40). However, invasion of the central nervous system is not frequently observed and it depends on the utilized TPA strain (41). In addition to rabbits, there are other species that are also susceptible to TPA (i.e. non-human primates, hamsters, guinea pigs, and mice). While only non-human primates and rabbits develop clinical signs similar to humans (40, 42), mice and other rodents may be useful to study bacteria dissemination (43).

The TPA genome is a circular chromosome of approximately 1138 kilobase pairs that contains 1041 predicted ORFs (28). Its genome is small compared to other pathogenic bacteria (28). The genome sequence confirms that TPA cannot synthetize *de novo* enzyme cofactors, fatty acids, tricarboxylic pathway enzymes, or nucleotides, even though it contains 57 ORFs encoding transport proteins and is able to use the glycolytic pathway (28). TPA lacks genes encoding superoxide dismutase, catalase, or peroxidase, which could explain its susceptibility to oxygen. Additionally, several genes have also been identified that are involved in the synthesis of motile proteins and lipoproteins (4, 8, 28). Genome comparison among pathogenic *Treponema pallidum* subspecies has shown that they are similar in size and structure, and differ less than 0.2%-0.4% in their genome sequence (44, 45).

## 3 Infection course and immune response

Syphilis is generally transmitted through sexual contact or from mother to child. Spirochetes gain access to the host through epidermal micro-abrasions or directly penetrating mucosal membranes (46, 47). Once TPA has entered the body, it adheres to epithelial cells and extracellular matrix and locally multiplies with an estimate rate of once every 30-33 hours (48, 49). *In vitro* binding studies have shown that laminin and fibronectin are among the anchor molecules involved in these interactions (50–53). Since TPA lacks cytotoxic toxins or other virulence factors (28), tissue destruction and chancre lesions are probably caused by the inflammatory response at the entry site (47). The initial immune response clears bacteria locally, and the primary stage lesions resolve spontaneously within 3-6 weeks. Meanwhile spirochetes spread throughout the body and the infection rapidly becomes systemic, triggering secondary syphilis. Afterward, spirochetes penetrate deeper tissues and induce the expression of inflammatory signals, promoting the migration of immune cells to the infected tissues (47). *In vitro* studies have shown that TPA can induce the expression of the adhesion molecules ICAM-1, VCAM-1 and E-selectin in endothelial cells (54–56). In addition, metalloprotease activity plays a major role in the penetration and dissemination of TPA through extracellular matrix and intercellular junctions (57, 58). In the final stage, infection and the associated immune response damage various organs (e.g. heart and brain), resulting in debilitating health problems (47).

**Figure 1:**
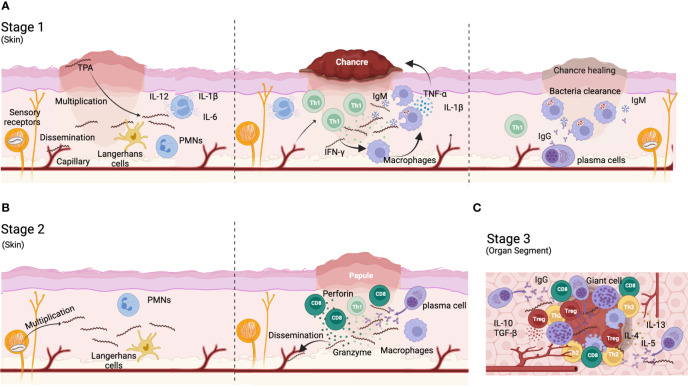
Immune response to TPA infection. **(A)** During primary syphilis, a local lesion called chancre appear in the site of infection. After bacteria entry, the microorganisms proliferate and disseminate to distal organs. Bacteria are phagocyted by polymorphonuclear cells (PMN) and dendritic cells that migrate to draining lymph nodes to activate CD4^+^ Th cells. Th1 T cells migrate to the bacteria proliferation foci and produce Th1 cytokines (IFN-γ) for recruiting and activate macrophages. M1 macrophages phagocyte bacteria and produce TNF-α and IL-1β that contribute to chancre development and eventually necrosis. IgM antibodies and complement contribute to bacteria opsonization favoring their removal by macrophages. **(B)** During secondary syphilis, TPA expands from reservoirs with systematic affectation. A typical rash appears in most cases. Anti-TPA humoral responses are mainly IgGs and plasma cells are visible in the infiltrate of secondary cutaneous lesions. CD8^+^ T-cells are more represented than CD4+ T-cells in lesion infiltrates. **(C)** Tertiary syphilis affects deep organs like brain and heart. A Th1/Th2 switch progressively occur and the immune response is down regulated by the presence of regulatory T cells. Plasma cells are well represented. Gummas can be developed in different locations (i.e., liver, skin, brain). Gummas are a granuloma-like structure characterized by a necrotic hyaline nucleus surrounded by immune cells (i.e. plasma cells, macrophages and giant cells) and fibroblasts.

TPA infection triggers a complex immune response that fails to control the spread of the bacteria and the progression of the disease. In primary syphilis, polymorphonuclear leukocytes (PMNs) are the first immune cells to infiltrate the infection site (59) (**Figure 1A**). These cells may contribute to the initial control of the infection by secreting anti-microbial peptides (60) and clearing spirochetes by phagocytosis (59, 61). However, the initial control of the infection is limited as bacteria dissemination occurs in virtually all cases. Besides PMN, dendritic cells (DCs) (**Figure 1A**) can also phagocyte whole or bacteria-derived fragments, thereby stimulating their maturation and antigen-presentation capacity. Thus, DCs increase the production of pro-inflammatory cytokines, such as IL-12, IL-6 and IL-1B, and upregulate the expression of CD54, CD83, CD80, CD86, and HLA-DR (62, 63). Mature DCs migrate to lymph nodes where they present treponemal antigens to T cells, inducing antigen-specific T cells responses. These T cells can be detected in secondary lymphoid organs of infected animals three days post-infection and progressively accumulate at the infection site (64–66), coinciding with maximal TPA burden (67, 68). Rabbits infected with TPA showed a rapid hyperplasia of T cell zones in secondary lymphoid organs (lymph node and spleen) (38, 66). Although Th1 CD4^+^ T cells dominate the T cell infiltrate (38, 61, 69)(**Figure 1A**), cytotoxic T cells (CD8^+^) can also be found in primary lesions, where their role remains unclear. CD8^+^ T cell may be required for the elimination of TPA reservoir inside non-phagocytic cells in early syphilis lesions (69–71). In this sense, perforin and granzyme are detected in early lesions, suggesting cytolytic activity (72). Furthermore, CD8^+^ T cells, as well as NK cells, could contribute to the secretion of interferon-γ (IFN-γ) (9, 73). Th1 cytokines can promote the migration and activation of macrophages (**Figure 1A**), whose number increase rapidly by day 10 after infection at the entry site (68). Activated macrophages phagocytose and destroy spirochetes (64, 74) until they are completely cleared from the infection site. Interestingly, this process can be enhanced by TPA opsonizing antibodies and IFN-γ, particularly through the Fc-Fcγ receptor interaction (73–77). However, other less efficient mechanisms, such as non-opsonic phagocytosis and active direct invasion, may also participate in TPA-macrophage interplay (78). In response to TPA infection, macrophages polarize to M1 phenotype and secrete proinflammatory cytokines (i.e. IL-1β, TNF-α, IL-12, and IL-15) (77, 79), which can ultimately promote necrosis and ulcer formation typical of primary chancre lesions.

Regarding the humoral response, anti-TPA antibodies can be detected as early as 6 days post-infection (80). However, the antibody response kinetics vary based on the target protein and TPA strain. For example, antibodies targeting Tpr Subfamily I and II rise between days 10 and 45 after infection (81). During primary infection, IgM dominates the anti-TPA humoral response, while IgG levels gradually increase (80, 82) (**Figure 1A**). Antibodies are not only involved in opsonization processes, they can also block bacteria dissemination and dermal lesions. Furthermore, the complement system is also involved in limiting treponemal activity, specially working in conjunction with antibodies (83–85). TPA accumulates sialic acid on its surface, making it resistant to complement lysis by the alternative pathway (86–89). Thus, classical complement activation pathway is key to bacterial clearance. This data suggest that the immune response generated against TPA during primo-infection is a delayed type hypersensitivity (DTH) immune response, where sensitized T cells play a major role. In fact, it has been hypothesized that syphilis prognosis depends on the balance between DTH and humoral responses. Thus, it has been proposed that a strong DTH immune response may be required to clear the infection, whereas latency may result from intermediate DTH responses. Tertiary disease would be related to a weak DTH and a strong humoral response (68, 90). Despite this, antibodies may be crucial for preventing infection (68, 91). Accordingly, rabbits receiving immune serum showed a delay in the appearance of lesions that were also less severe (92). Further, patients who have been previously treated for syphilis remain susceptible to reinfection because they do not develop an effective anti-TPA humoral response (68).

Both cellular and humoral responses are maintained for months after clearance of primary syphilis lesions (39, 93, 94), and their magnitude correlate with the persistence of TPA as latent infection (95–98). From this state, relapse of active infection can occur leading to secondary and tertiary syphilis. Secondary syphilis manifests as inflammatory cutaneous affection that differs in histological appearance from primary syphilis. The histology of secondary syphilis is variable but, in general, lymphocytes, macrophages, and plasma cells are commonly present in secondary lesions whereas polymorphonuclear and eosinophilic infiltrates are found in a smaller proportion (99–101) (**Figure 1B**). Furthermore, CD8^+^ T cells are highly represented (**Figure 1B**) (99). Contrary to primary syphilis, IgGs dominate the anti-TPA humoral immune response during secondary syphilis (**Figure 1B**) (82, 102, 103). Moreover, whereas IgG1 is the main IgG subclass in primary syphilis, both IgG1 and IgG3 subclass can be equally found in secondary syphilis (104).

Finally, tertiary syphilis is characterized by a multiorgan affection and the development of gummas in different tissues (such as liver and skin). Gummas are granuloma-like structures characterized by a necrotic nucleus surrounded by macrophages, giant multinucleate cells, lymphocytes, and plasma cells (68) (**Figure 1C**). Neurosyphilis is characterized by an increase in the number of CD8^+^ T cells in blood (105). Moreover, a switch in the production of Th1 to Th2 cytokines is observed during the evolution of the disease. While Th1 cytokines are mainly detected during primary syphilis, Th2 cytokines increase in late states of the disease (106) (**Figure 1C**). This correlates with cell depletion in the diffuse cortex, and a follicular hyperplasia in lymph nodes with plasma cells accumulation within the interfollicular areas during tertiary syphilis (68). These changes in the immune response during syphilis are closely related with disease progression.

## 4 Immunization studies and vaccine strategy

Despite public health campaigns and the availability of efficacious treatment, syphilis prevalence has increased worldwide in the last decade (17), suggesting the need for additional measures to control the transmission of the infection. The development of a syphilis vaccine could be a valuable tool. However, after several decades of research, an effective vaccine for syphilis remains elusive. A variety of strategies have been tested, including inactivated bacteria and subunit recombinant proteins, even though with limited success. Interestingly, Miller et al. demonstrated protection of rabbits against TPA infection following 60 immunizations with γ-irradiated bacteria for 37 weeks (107). Although this experimental strategy is far from being applicable to humans, it serves as a proof of concept that a syphilis vaccine is feasible. In addition, human challenge studies performed in the 50’s showed that latently infected patients were resistant to reinfection with a heterologous TPA strain (108). Accordingly, Marra and colleagues reported that previous syphilis infection may attenuate the manifestation of subsequent TPA infection (109). Thus, those individuals that experimented three or more episodes of syphilis were more likely to develop latent early syphilis after subsequent infections. These studies indicate that it will take a long time to establish a protective immunity against TPA, further emphasizing the low immunogenicity of this pathogen.

Until 2018, one of the main handicaps in the development of a syphilis vaccine was the inability to grow the bacteria *in vitro (* 34). This technical limitation shifted the focus of most studies towards recombinant OMPs (110). Although none of these proteins provided complete protection against *in vivo* TPA challenge, promising results were observed; namely, the induction of a strong humoral response, less ulcerative lesions, faster recovery of lesions, or inhibition of bacterial dissemination to distal organs. These studies provided meaningful knowledge about OMPs, immunization regimens, and potential vaccine targets. The advancement of bioinformatic tools allowed the identification of additional putative OMPs, as well as the prediction of structure models and B cell epitopes (111, 112). The employment of newly-developed approaches (e.g. genetic engineering), and refined bioinformatic tools enable to delve further into OMPs knowledge, benefiting future vaccine studies (113, 114).

## 5 The outer membrane proteins

One of the major features that differentiate TPA from other bacteria is the paucity of surface-exposed OMPs (24–26). It has been calculated that the density of OMP in TPA is approximately 100-fold less than that in *E. coli (* 22). The identification and characterization of TPA OMPs has been challenging due to the inability to genetically manipulate and cultivate TPA *in vitro* (until recently) (34), and the fragility of its OM, which generated a strong controversy in some studies. The intrinsic properties of the OM (i.e lack of LPS and low density of proteins) makes it can be easily damaged by common experimental manipulations (e.g. centrifugation, resuspension, or using low concentration of non-ionic detergent) (22, 26). OM disruption leads to potential exposure of lipoproteins that are normally present in the periplasmic space and cytoplasmic membrane, and could erroneously pinpoint proteins as OMPs. Due to these technical limitations, the identification of OMP was often based on prediction of their sequence and structure or by comparison with functional orthologs from other species (111). Thus, OMPs can be classified in three main groups according to their putative functions:

### 5.1 Transport function

Since nutrient import and toxics efflux are crucial for bacteria surveillance, several TPA OMPs have been involved in transport function.

#### 5.1.1 Tpr protein family

The *Treponema pallidum repeat* (Tpr) family of proteins include 12 members that are divided into three subfamilies according to their amino acid composition: subfamily I (TprC, D, F, I), subfamily II (TprE, G, J) and subfamily III (TprA, B, H, K, L). Among them, subfamily I and TprK (subfamily III) are the most widely studied. Regarding Tpr protein location, sequence analysis predicted that TprB, TprC, TprD, TprE, TprG, TprH, TprI, TprJ, TprK and TprL could be located in the OM (22). Supporting this prediction, a putative cleavable signal peptide was identified in most of the Tprs superfamily members, including TprC, D, F, I, E, G, J, A, B and TprK proteins (111, 115).

The Tpr family is related to the major outer sheath protein (MOSP) of *Treponema denticola (* 115), a surface-exposed protein with adhesive and porin function (116–118). Most Tprs include three main domains: 1) a N-terminal domain related to the N-terminal domain of *Treponema denticola* MOSP, 2) a central region, and 3) a C-terminal domain related to the C-terminal domain of *Treponema dentiola* MOSP, that is lacking in TprF and TprA proteins. Recently, the presence of short molecular recognition features (MoRFs) was predicted in most Tprs. These motifs may interact with specific proteins and undergo disorder-to-order transition upon binding (111). Sequence analysis has shown that the N- and C-terminal domains of the Tpr proteins from subfamily I and II are relatively conserved, whereas central domains are variable in length and sequence (6). Terminal domains present amphipathic regions and flank hydrophilic regions of the central domains. According to Hawley et al., the N- and C-terminal domains from all Tprs adopt a β-stranded structure, while the central regions may be mostly α-helices (111).

Structural analysis of the subfamily I has shown that these proteins form a β-barrel trimeric channel. However, unlike classical porins, which the entire polypeptide forms a β-barrel, Tpr subfamily I possesses bipartite topology. The C-terminal domain forms a β-barrel structure and is surface-exposed, while the N-terminal and central domains anchor the β-barrel into the peptidoglycan sacculus of the periplasmatic region (119, 120). Accordingly, while the expression of TprC/D in *E. coli* is surface-exposed, TprF lacks the C-terminal domain, and therefore, it is entirely periplasmatic (22). The immunodetection of Tpr proteins by electron microscopy using purified rabbit anti-Tpr I immunoglobulins, confirmed the presence of Tpr proteins in OM and periplasmic spaces, which could be due to the bipartite structure previously described or because the anti-Tpr I antibody recognized proteins with distinct location (121). More recently, Hawley et al., confirmed that the bipartite membrane topology identified in subfamily I is common to all Tprs with three full domains (111). Based on sequence and structure analysis, it has been shown that the Tpr family may be involved in the import of small soluble molecules. In this sense, the integration of some Tpr proteins in liposomes increased their permeability (119, 120). Therefore, changes in protein expression and/or variation of their channel-forming β-barrel region could be used to adapt TPA nutritional requirements to the environment. Accordingly, the expression of Tpr proteins can vary among strains, and in the same strain overtime (122), which may explain why the anti-Tpr humoral response differ among TPA isolates (81). This observation suggests the existence of mechanisms that can regulate the expression of these genes. Thus, a hypervariable homopolymeric guanosine (poly-G) tract and a cAMP receptor protein (CRP) binding motif have been identified upstream of the transcription start site of the Tpr subfamily II and III (123–125). In fact, Tp0262 (a TPA CRP homologue) can bind to these promoters and regulate the surface expression of these proteins during infection (123). As a result, the specificity of the humoral response elicited against Tpr proteins differ among TPA isolates (81).

Of the Tpr superfamily, TprK is one of the best characterized proteins. It has been previously showed that recombinant TprK is a monomeric porin by negative-staining electron microscopy (126). Anti-TprK antibodies can be detected early during infection (81). However, TprK exhibits sequence variation that contributes to immune escape. Thus, several alleles have been identified among TPA strains (127, 128). Moreover, this protein shows seven variable domains whose sequence changes by non-reciprocal recombination, mainly with regions downstream of the TprD gene (127, 128), a mechanism that is enhanced under immune pressure (129, 130). The existence of this mechanism could explain why TprK variability was higher in TPA isolates from patients affected with secondary syphilis than those with primary syphilis (128). However, TprK genetic variation can also occur in the absence of immune pressure (131). Interestingly, it has been reported that humoral response *in vivo* targets variable regions, while cellular response recognizes conserved TprK epitopes (132, 133). Moreover, animal challenges using TPA with TprK homologous to the one used in immunization regimen show better protection than those animals challenged with a strain expressing heterologous TprK (132). Despite that, there are controversies on the role of anti-TprK antibodies, and TprK protective efficacy as immunogen and its location. Centurion-Lara and colleagues described that TprK is located on the OM, and TprK-specific antibodies had opsonization capacity (115). Interestingly, rabbits immunized with this protein did not develop ulcerative lesions. These lesions healed faster than those observed in unvaccinated control animals and contained fewer spirochetes, when analyzed by darkfield microscopy (115). Additionally, Morgan et al. identified the N-terminal region of TprK (37-273 amino acids) and, to a lesser extent, the C-terminal portion (349-478 amino acids), as the parts of the protein that induced the previously-described protective immune response (134). Conversely, Hazlett and colleagues described that TprK was located in the periplasmic space, anti-TprK antibodies lacked opsonization activity, and immunization with recombinant TprK did not induce a protective immune response nor TprK sequence variation (135). Further investigation would be needed to clarified these discrepancies.

Regarding the immunity elicited against other Tprs members, anti-TprI antibodies were detected in 98% of syphilis-affected individuals. These antibodies mainly targeted the conserved N-terminal region. The lack of reactivity was associated with early infection (121). Remarkably, experiments performed in rabbits showed that TprI immunization did not protect rabbits from infection (136). However, cutaneous lesions did not ulcerate and healed faster than those generated in unvaccinated animals. This protein elicited strong humoral and cellular responses mainly targeting the conserved N-terminal region of the protein during TPA infection (81, 136). Not surprisingly, immunization with the conserved N-terminal region of TrpF, which is also common to all subfamily I members, did not protect rabbits from TPA infection after challenge. However, TprF vaccination attenuated lesion development, prevented ulceration, and reduced bacterial burden in skin lesions (136). In regards to TprC and TprD, epitope mapping has shown that the humoral response detected in rabbit sera is directed against exposed loops (112). Moreover, the N- and C- terminal regions contain the most reactive epitopes. Surprisingly, Anand et al. showed that sera from infected individuals do not recognize TprC, while TPA-infected rabbits develop antibodies with opsonization activity (119). One explanation could be the low expression of TprC and the different magnitude of the humoral response found among various syphilis strains and subspecies.

#### 5.1.2 Tp0515

Tp0515 is a structural ortholog of LptD protein, which is part of a multiprotein complex involved in LPS trafficking to the OM in Gram-negative bacteria (22). Thus, since LptD is embedded in the OM, Tp0515 is inferred to be an OMP. However, sequence analysis of TPA genome failed to identify LPS biosynthetic pathway (28). Notably, orthologous proteins for several components of the LPS transport pathway have been found in TPA (111), suggesting that even without LPS, these proteins could be involved in translocation of other cellular products to the OM, as glycolipids (22). Tp0515 structural modelling found that this protein is a 26-stranded β-barrel embedded in OM with exposed extracellular loops (111). Moreover, Hawley et al. identified B cells epitopes inside these extracellular loops using bioinformatic analysis (111). However, the immunogenicity and capacity of this protein to induce protective immune response still need to be empirically demonstrated.

#### 5.1.3 Tp0126 and other OmpW/AlkL orthologs

Four proteins that are orthologs of OmpW (Tp0126 and Tp0733) and OprG (Tp0479 and Tp0698) have been identified in TPA (111). Both *E. coli* OmpW and *Pseudomonas aeruginosa* OprG proteins are members of the OmpW/AlkL family of proteins, which have been suggested to be involved in bacterial adaption to environment stress and nutrient acquisition (137, 138). OmpW and OprG have an eight-stranded β-barrel architecture and form a peculiar hydrophobic channel, which allows the transport of hydrophobic molecules directly to the membrane, bypassing the hydrophilic periplasmatic space (111, 139). Thus, Tp0126 could be involved in fatty acid transport (unable to be synthesized by TPA alone) (140). Interestingly, this protein is highly conserved among TPA strains. Its transcription is regulated by the presence of guanidine homopolymers of various length located upstream of its promoter, which is consistent with phase variation (141).

Remarkably, Tp0126 may show low immunogenicity. Anti-Tp0126 antibody levels in serum samples from patients with latent syphilis were lower than those targeting Tp0574, a highly expressed and immunogenic TPA protein (141). In addition, anti-Tp0126 antibodies were detected in Tp0126-immunized rabbits only after the third boost, highlighting the potentially low immunogenicity of this protein (141). Interestingly, these antibodies targeted Tp0126-external loops and were able of opsonize TPA. However, immunization with Tp0126 showed poor protective capacity, if any, in rabbits. In fact, treponemes isolates from lesions of immunized and challenged rabbits showed lower transcription levels of Tp0126, which was associated with a longer polyG region (≥ 9 G’s), when compared to treponemes obtained from the challenge inoculum (141). These results suggest that the expression of Tp0126 may be reduced during infection, which could explain the absence of protection observed in immunization and challenge experiments.

Like Tp0126, other members of the TPA OmpW/AlkL ortholog group (Tp0733, Tp0479, and Tp0698) (111) are predicted to form a membrane-channel and being involved in transport of small molecules. Hawley et al. predicted multiple surface-exposed B cell epitopes in their extracellular loops, although the density of these epitopes differs from one to another protein (111). Future experimental studies with these OMPs are envisaged to confirm their *in silico* predictions, and to inform about potential immunogenicity and capacity of inducing protective immune responses.

#### 5.1.4 FadL-like proteins

Due to the low OM permeability of Gram-negative bacteria, these bacteria have an OM protein machinery involved in the uptake of long-chain fatty acids, such as the FadL (Long-chain fatty acid transport protein). TPA is unable to synthesize long-chain fatty acids (28). However, since TPA OM is more permeable to fatty acids than other Gram-negative bacteria (142), membrane diffusion may be one of the mechanisms that this microorganism uses to uptake these molecules. Nevertheless, five FadL orthologs (Tp0548, Tp0856, Tp0858, Tp0859, and Tp0865) have been identified in TPA, suggesting that passive diffusion of fatty acids is not the only mechanism (111). Structural modelling of these orthologs predicted that all TPA FadL-like proteins form a 14-stranded β-barrel with N-terminal in the barrel lumen (111). Moreover, all five proteins contain one or more predicted B cell epitopes (111). Interestingly, Delgado et al. recently described the presence of IgG antibodies and IgG^+^ B cells in TPA-infected rabbits that recognized the extracellular loops 2 and 4 from Tp0856 and Tp0858 (143), indicating that these proteins could be useful in vaccine design.

#### 5.1.5 TolC-like proteins

TolC is an OM protein from *E. coli*, which is part of an efflux pump complex involved in the removal of toxic substances (144). To date, four TolC orthologs (Tp0966, Tp0967, Tp0968, and Tp0969) have been described in TPA (145). Structural modelling of these proteins identified that they have a TolC-like topology based on four β-strands with two large extracellular loops and six α-helices (111). Furthermore, Hawley et al. predicted that all four TolC-like TPA proteins, particularly Tp0969, enclose surface-exposed B cells epitopes (111). Thus, these predicted OMPs could be targeted by the immune system. However, further *in vivo* analysis of their immunogenicity is required to determine their potential in vaccine development.

#### 5.1.6 Treponema rare outer membrane proteins (TROMPs)

TROMP family includes three proteins: TROMP-1 (31 kDa), TROMP-2 (28 kDa) and TROMP-3 (65 kDa) (146). TROMP-1 (also called TroA or Tp0163) was firstly described as a surface-exposed protein with porin-like properties (147). However, it was later identified to be a periplasmatic metalloprotein anchored to the cytoplasmatic membrane (148–150). TROMP-2 (also called Tp0663) was localized on the OM when it was expressed as recombinant protein in *E. coli*. However, the isolation of TROMP-2 from *E. coli* OM showed low porin insertional events, casting doubts on its porin function (29). Interestingly, both TROMP-1 and TROMP-2 were targeted by antibodies present in sera from infected rabbits and humans (146). In fact, TROMP-2 could be a potential candidate for serodiagnosis of all syphilis stages (151). TPA challenge experiments performed in Tp0663-immunized rabbits showed partial protection, with high titers of anti-Tp0663 antibodies. The authors observed attenuated lesions with an increase cellular infiltration. Importantly, no bacteria dissemination to distal organs was detected in immunized animals (152). Of note, TROMP-3 is expressed at a lower concentration than the other two members of this family, and its function and structure remain unknown (29).

### 5.2 Adhesion function

Adhesion to cells and extracellular matrix is crucial to the establishment of infection and the dissemination of TPA.

#### 5.2.1 Tp0136

Tp0136 has been identified as a fibronectin-binding TPA OMP that binds more efficiently to cellular than plasma fibronectin (50, 51). This selective binding involves different domains of this protein. Thus, the conserved N-terminal region is mainly responsible for binding to plasma fibronectin. Residues in the C-terminal end, and also in the central portion of the protein, participate in binding to the cellular form of fibronectin (51). Interestingly, Tp0136 shows sequence variability among TPA strains, and its transcription is regulated during infection (50, 51). The interaction of Tp0136 with fibronectin expressed on cell surface can promote bacteria attachment to tissues, facilitating body dissemination and favoring the colonization of distal endothelial tissues, central nervous system, or placenta (153).

In addition to its adhesion function, Tp0136 may also play an important role in chancre healing by promoting fibroblast and microvascular endothelial cell migration, and the activation and aggregation of platelets (154).

Immunization and challenge experiments performed in rabbits using recombinant Tp0136 produced in *E. coli* showed a delay in lesion ulceration but not in their development, indicating no protection against infection (50). Remarkably, sera obtained from immunized animals reduced the attachment of the bacteria to fibronectin, although at lower levels than sera obtained from unvaccinated and infected rabbits (50). Interestingly, Xu et al. showed that rabbits immunized with Tp0136 elicited high levels of antigen-specific antibodies, attenuated lesion development with increased cellular infiltration, and limited treponemal dissemination to distant organs (152). Although there are discrepancies between both studies that could be explained by differences in adjuvant, immunization schedule, and the amount of live inoculated bacteria, these results suggest that antibodies developed against Tp0136 might be protective. However, the fact that antibodies elicited during experimental infection blocked more efficiently TPA binding to fibronectin than vaccine-induced antibodies elicited against Tp0136, suggest that, during vaccine design, other specificities should be also targeted to more efficiently block bacteria from binding to the extracellular matrix (51).

#### 5.2.2 Tp0155

It has been described that Tp0155 preferably binds to fibronectin matrix and, therefore, it might be involved in the dissemination of TPA (155). Tp0155 shows two lysin motifs (LysM) domains in its N-terminal portion that can play a major role in binding to fibronectin and peptidoglycans, and a M23 peptidase domain in the C-terminal region, which might show peptidoglycan lytic activities (156).

There is some controversy regarding Tp0155 surface location. Inhibition assays showed that recombinant Tp0155 reduced live TPA binding to fibronectin-coated slides, suggesting that Tp0155 is expressed on the OM (155). This result was in line with the fact that TDE2318, a fibronectin-binding protein with high homology to Tp0155, was found in the surface of *T. denticola* according to immunofluorescence microscopy data (156). However, immunofluorescence studies using agarose microencapsulated treponemes showed that Tp0155 is not exposed on the OM of TPA (157). According to this observation, Tp0155-immunized rabbits, which elicited high antigen-specific antibody titers, were not protected against an intradermal challenge with live treponemes (157). Thus, further investigation will be needed to determine whether Tp0155 would be a bona fide OMP or not.

Remarkably, studies performed with human sera showed that approximately 70% of syphilis patients did not show antibodies against Tp0155, indicating that the expression of this protein or its immunogenicity may be low in natural infection (157, 158).

#### 5.2.3 Tp0435

Tp0435 is a highly immunogenic 14 kDa lipoprotein with adhesin function, also known as Tp17. To identify the location of Tp0435 in TPA, Chan and colleagues performed a study that analyzed the Tp0435 surface expression in TPA and Tp0435-transformed *B. burgdorferi* cells (159). The authors concluded that Tp0435 may be post-translationally modified, generating several variants that can be differentially located between the surface and the periplasmic space. Contrarily, Cox et al., did not find Tp0435 surface-exposure evidences (160). Structurally, Tp0435 encompasses eight-stranded anti-parallel β-barrel with a basin-like domain located at one end (161). According to Chan et al., Tp0435 favors the attachment of the spirochetes to mammalian cell lines (159). Interestingly, human sera from infected patients are reactive against Tp0435 (158), and immunization of rabbits with a Tp0435-expressing *B. burgdorferi* strain induced a strong immune response against Tp0435. However, this immune response failed to protect from infection or lesion development after experimental challenge (162). Thus, further studies may be needed to determine the location of Tp0435 and its potential as vaccine candidate.

#### 5.2.4 Tp0483

Tp0483 can bind both extracellular matrix and soluble fibronectins (155). The C-terminal portion of Tp0483 (179-374 amino acids) also showed reactivity against laminin (163). Little is known about the structure of Tp0483. However, a peptide encompassing 316-333 amino acids inhibited binding of Tp0483 to fibronectin, suggesting that this peptide, or a near region of Tp0483, should be responsible for binding to adhesive glycoprotein (164). Similar to Tp0155, Cameron et al. indirectly suggested that Tp0483 was an OMP (155). Contrarily, Tomson and colleagues did not find evidences of the Tp0483 OM location. Even though rabbits immunized with this protein elicited high antibody titers, the authors did not detect protection after challenge (157).

#### 5.2.5 Tp0750

Tp0750 binds to fibrinogen and the fibrinolytic receptor complex protein Annexin A2. Tp0750 shows serine metalloprotease activity, and it is able to degrade fibrinogen and fibronectin, inhibiting the coagulation cascade, but cannot degrade fibrin clots (57). Structurally, Tp0750 shows metal ion-dependent adhesion site (MIDAS)-containing von Willebrand Factor type A domains (region V29-T147). The MIDAS domains bind to primary calcium. This protein, as well as Tp0751 (see section 5.2.6), are co-transcribed and might coexist as heterodimeric complex. However, unlike Tp0751, Tp0750 shows a limited laminin binding capacity (57). Sequence analyses of both proteins have shown that Tp0750 is highly conserved among pathogenic treponemes species, whereas Tp0751 is less conserved, except for the most invasive treponemes species. Interestingly, Tp0750 and Tp0751 orthologs from the less invasive treponemes species do not bind or degrade host proteins, which strengthen the idea that both adhesion function as well as degradative capabilities are important to syphilis progression (165).

#### 5.2.6 Tp0751

While there is a degree of agreement in regards to the structure of Tp0751, there are discrepancies regarding its function, location, and the relevance of the immune response elicited against this protein. From the structural point of view, Tp0751 presents a bipartite topology with a disorganized N-terminal region that is joined through an α-helix domain to a C-terminal portion arranged in eight anti-parallel β-sheets (52, 166).

It has been previously described that Tp0751 binds to different forms of laminin in a dose-dependent manner, and can work as vascular adhesin, promoting bacterial dissemination (53, 167). Additionally, Tp0751 showed metalloprotease activity, and can degrade fibrinogen and laminin, promoting clot dissolution, and favoring bacterial dissemination (58). However, Luthra and colleagues showed that Tp0751 is located in the periplasmic space and its main role is binding to small molecules along the barrel rim, such as hemes (166).

While Cameron and colleagues described that Tp0751 is recognized by antibodies in patient and infected rabbit sera (163), and these antibodies can opsonize the bacterium, Luthra et al. observed opposite results. In their study, Tp0751 induced a weak antibody response in infected human and challenged rabbits, and anti-Tp0751 antibodies lack opsonization capacity, probably due to the low levels of Tp0751 expression on the OM (166). Immunization and challenge experiment in rabbits also provided contradictory results. Lithgow and colleagues showed that Tp0751-immunized animals showed attenuated lesions with low bacteria burden. Although immunization did not prevent infection, it successfully inhibited bacteria dissemination (168). Conversely, Luthra et al. showed no protection against local or disseminated infection following intradermal TPA challenge in Tp0751-immunized animals (166). Therefore, further research will be needed to clarify these discrepancies.

#### 5.2.7 Tp0954

Tp0954 has been recently described as a surface lipoprotein, and may have a major role in congenital syphilis (169). According to its structure, this protein is predicted to have a tetratricopeptide repeat (TPR) structural motif with tandem α-helices. Moreover, Tp0954 sequence is conserved among several TPA strains (e.g. Nichols, Chicago, Mexico A and Amoy) (169). Primus et al. also described that Tp0954-transformed *B. burgdorferi* B314 bacteria, which is known to be a poorly adherent strain, gained binding to mammalian epithelial cell lines, including a human placental cell line [i.e. BeWo (CCL-98)] (169). Unlike other TPA adhesins, Tp0954 can mediate adhesion and bacteria dissemination through binding to glycosaminoglycans present in human placenta, such as dermatan sulfate, heparin, and heparan sulfate, which are components of the extracellular matrix (169). Dermatan sulfate is associated with fetal blood vessels and syncytial surface, whereas heparan sulfate is located in trophoblast layers (170–173). Thus, it is possible that Tp0954 might facilitate congenital infection through binding to placental glycosaminoglycans.

### 5.3 Other functions

A number of proteins in the OM of TPA are not related with neither transport or adhesion function.

#### 5.3.1 Tp0326

Tp0326 (or Tp92) shows homology with BamA orthologs from other Gram-negative bacteria. BamA-like proteins are characterized by a bipartite structure with a β-barrel domain located in the C-terminal portion, and at least one polypeptide-transport-associated (POTRA) domain in the N-terminal end. Particularly, Tp0326 was predicted to contain five N-terminal POTRA and one C-terminal β-barrel domains (174, 175). According to Desrosiers et al., the N-terminal POTRA domains are periplasmic, whereas the C-terminal β-barrel is embedded in the OM forming a pore (175).The N-terminal end can bind to multiple periplasmatic proteins through POTRA domains, establishing a protein complex that is crucial for membrane synthesis. As other BamA-like proteins, Tp0326 is suggested to be part of a protein machinery involved in the translocation and insertion of proteins from the periplasmatic space to the TPA OM (175). Interestingly, human antibodies are mainly directed against the Tp0326 periplasmic segment, while rabbit antibodies target both N-terminal and C-terminal regions (175). Indeed, Luthra et al. found that POTRA domains are immunodominant over the β-barrel domains in both rabbits and humans (174). In addition, these authors described that the Tp0326 central pore exposes eight loops to the extracellular space. Among them, loop 4 is specially targeted by antibodies with opsonizing capabilities from infected rabbits and humans with secondary syphilis (174). By contrast, Tomson and colleges failed to detect the expression of Tp0326 on the OM of treponemes encapsulated within agarose gel microdroplets using indirect immunofluorescence (157). In addition, they did not observe protection in Tp0326-immunized animals after TPA challenge. Conversely, Cameron et al. showed that, although all Tp0326-immunized rabbits were infected after challenge, there was some degree of protection in the vaccinated animals that had high antigen-specific antibody titers (176).

#### 5.3.2 Tp0257

Tp0257 (or GpD) was identified using a treponema genetic expression library and opsonized rabbit immune sera. This protein presents homology with *H. influenzae* GplQ protein, a glycerolphosphodiester phosphodiesterase protein, which is partially expressed on the surface (177). Thus, it is possible that Tp0257 may be an OM protein. Accordingly, Cameron et al. reported indirect evidences of its surface location by immunoblot analysis of TPA lysates. The authors observed positive staining in the preparation that included the OM, but not in the OM-removed TPA lysate fraction (178). Moreover, GpD was isolated from OM preparations (179). Interestingly, rabbits immunized with recombinant Tp0257 exhibited a reduction in lesions development and bacterial proliferation after TPA challenge (178). Contrarily, Shevchenko et al., showed that Tp0257 could be a periplasmic protein, since they did not identify surface-exposed evidences by multiple assays, and Tp0257-immunization failed to protect rabbits from TPA challenge (180). More recently, a DNA vaccine encoding a Tp0257-IL-2 fusion protein showed a decrease in ulcerative lesions, as well as, in the number of lesions containing TPA (181). The inclusion of IL-2 in the vaccine enhanced anti-GpD humoral responses and the levels of IFN-γ. Remarkably, rabbits immunized with a Tp0257-IL2 DNA prime and intranasal boost with recombinant protein adjuvanted with CpG-ODN induced mucosal and systemic immunity, and showed a faster lesion recover after TPA inoculation (182).

Interestingly, Tp0257 is conserved among TPA strains, as well as in *Treponema pallidum endemicum* and *Treponema pallidum pertenue (* 183). These results make this protein an attractive immunogen candidate for vaccine design.

#### 5.3.3 Tp1038

Tp1038, also known as TpF1 or antigen 4D, is a bacterioferritin with ferroxidase activity playing a major role in iron uptake (184). Radolf et al. showed that Tp1038 is surface-exposed since sera from Tp1038-immunized rabbits was reactive to immobilized TPA in the presence of complement (185).

Tp1038 is able to induce diverse immune responses. For example, this protein might be involved in the proinflammatory response during primary syphilis, since it can activate the inflammasome complex in monocytes, and thereby, induces the release of IL-1β, IL-6, and TNF-α (186, 187), which could result in tissue damage associated with this stage. However, Babolin et al. described that Tp1038 may also drive a T regulatory response (186). CD4^+^ CD25^+^ Foxp3^+^ T cells from patients with secondary syphilis produce TGF-β under Tp1038 stimulation, and Tp1038-stimulated monocytes produce IL-10 and TGF-β, two cytokines linked to Treg cell differentiation (186). Therefore, TpF1 might be a virulence factor involves in the persistence of TPA infection through the downregulation of the immune response (186). Regarding the humoral response, anti-Tp1038 antibodies are detectable in all syphilis stages (186, 188). Finally, Pozzobon and colleagues identified that patients with tertiary syphilis have Tp1038-specific T cells, which stimulate monocyte and human umbilical vein endothelial cells to produce tissue factor, IL-8, and CCL-20 (189). These cytokines are involved in angiogenesis, thus Tp1038 may also be implicated in tertiary syphilis, in which vascular inflammation and angiogenesis characterize the lesions of this stage (189). Thus, Tp1038 is an interesting target for vaccine development since it is engaged in all syphilis stages.

## 6 Immune evasion strategies

**Figure 2:**
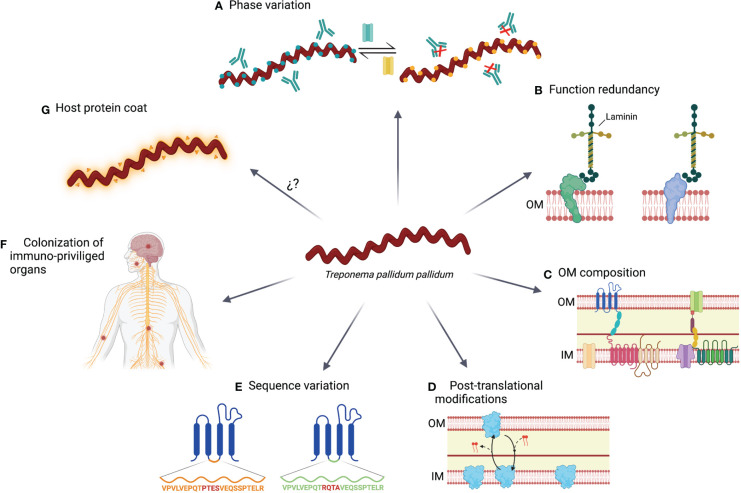
Immune evasions mechanisms use by TPA. **(A)** During its adaptation to environment changes, including the immune system pressure, TPA modulates the expression of certain genes by a switch on/off mechanism. **(B)** Linked with the prior mechanism; function redundancy ensure that bacterial homeostasis is not compromised by changing the expression of proteins with vital functions. **(C)** OM of TPA is characterized by a paucity of protein compare with the inner membrane (IM), the lack of LPS and the presence of anti-inflammatory phospholipids (i.e., phosphatidylserine and phosphatidylcholine). **(D)** Some proteins experience post-translational modifications that result in variants with different location preference. **(E)** Proteins can accumulate sequence variations by point mutation, gene conversion or recombination. **(F)** TPA can persist in immune privileged organs, such as central nervous system or nerve fibers, and prolong their survival by remaining hidden to the immune system. **(G)** TPA may be coated by host serum proteins, reducing its immunogenicity.

While an immune response is triggered against TPA, it does not protect against bacterial dissemination and disease progression to the second or third stage. In addition, reinfections are quite common, with an incidence around 5-22% (190–192), indicating that TPA developed some mechanisms to evade the immune system (**Figure 2**).

### 6.1 Phase variation or switch on/off

TPA can modulate the expression of several OMPs during its life cycle (122). However, the mechanisms controlling the expression of those proteins are not well characterized. In the case of subfamilies I and II of Tpr proteins, guanosine homopolymer (polyG) repeats have been identified upstream of the transcriptional start site. The length of the polyG sequences differ among treponemal isolates and modulate the expression of Tpr proteins at the transcriptional level (124). PolyG signals have also been found in other OMPs, such as the Tp0126 protein (140). Phase variation based on hypervariable homopolymeric repeats have been previously reported in several other bacteria such as *Neisseria meningitidis*, *Helicobacter pylori*, or *Haemophilus influenzae*, and it is crucial for the survival of pathogens facing host immunity (193–195). Moreover, other transcriptional regulatory mechanisms have been identified, such as the G4FS cis-acting DNA elements, which form guanine-quadruplexes and induce recombination and gene conversion of Tp0136 gene (196). Using these mechanisms, TPA can modify its OMP repertoire to adapt to the environment conditions and evade the immune response. Consequently, the specificity of the humoral response against Tpr proteins may vary between animals or humans that are infected with the same or different TPA strain, thereby supporting the existence of a variable repertoire of OMPs (81).

### 6.2 Functional redundancy

Several OMPs present similar functions in transport or cell adhesion. Functional redundancy may be advantageous since the replacement of one protein by a functional homologous can guarantee that phase variation and post-translational modifications will not adversely affect cell homeostasis (22). As an example, *Salmonella* has been reported to have two distinct flagellin genes, fliC and fliB, with the same function but different structure. By switching their expression from one flagellin protein to the other, the immune system becomes ineffective against assembled flagella (197).

### 6.3 Outer membrane composition

TPA is characterized by a low number of OMPs, which may encompass an advantage by limiting the number of exposed antigens to the immune system. Moreover, the spacing distribution of these proteins may promote that antibodies bind to them just with one arm, reducing the avidity of the interaction. This mechanism has been previously described in Human Immunodeficiency Virus (HIV) infection (198), which also presents low density of Envelope glycoprotein on the virion surface. In HIV, polyreactivity of neutralizing antibodies may increase binding avidity by heteroligation with membrane components (198). However, the development of these antibodies could be limited by tolerance mechanisms (199). Whether this phenomenon is also related to TPA immunity requires further investigation, but it could partially explain the low antibody efficacy at controlling infection. Since the OM lacks LPS (27, 28), and it is enriched in anti-inflammatory phospholipids (e.g. phosphatidylserine and phosphatidylcholine), these characteristics may also hamper the development of an efficient immune response (200, 201).

### 6.4 Post-translational modifications

Post-translational protein modifications play a crucial role in different processes of prokaryote cells, including persistence and virulence (202). These modifications have been found in the TPA proteome. For example, there are several variants of Tp0435 that may coexist and were generated by palmitoylation (159). Palmitoylation consists in the addition of lipid chains to terminal cysteine residues. This process has been previously reported in other bacteria and contributes to their infectivity and recognition of the immune system (203). Tp0435 variants may be located on the surface or into periplasmatic spaces, being the latter one the most abundant (159), which could explain why there is no consensus about the Tp0435 location. In fact, this protein has been the first description of post-translationally modified protein variants in TPA. However, other OMPs could be also susceptible to post-translational modifications.

### 6.5 Sequence variation

Genetic variation of several OMPs has been described in TPA, including laboratory-adapted or primary isolated strains (127, 129, 204). Some of these variations have been reported in the Tpr family, including TprC, TprD, TprG and TprK proteins (127, 204, 205). Interestingly, TprK accumulates sequence variation in variable regions (particularly in V6) by non-reciprocal recombination with silent cassettes, which is enhanced under immune pressure (129, 130). Additionally, several *tprk* alleles have been identified in different TPA strains (127), which contributes to increase the variability of this protein. Besides Tpr proteins, others OMPs, such as Tp0326 (BamA), also show genetic variants (204, 205). This protein has accumulated mutations on the extracellular hydrophilic loops predicted to contain B cell epitopes (204). Sequence variation was also confirmed in FadL orthologs proteins ranging from fully conserved Tp0856 to deeply variable Tp0548, as well as in Tp0136, Tp0868, Tp0966 and Tp0967 (206–208).

### 6.6 Colonization of immune privileged organs

TPA can colonize distal immune privileged organs, such as the central nervous system, placenta, or eyes. In these tissues, the action of the immune system may be limited, contributing to the persistence of this pathogen (209–211). In fact, studies performed using the rabbit model showed that TPA can be detected in nerves early after infection (68).

### 6.7 Host proteins coat

Similarly to other bacteria; for example, some streptococcal species (212), TPA immune evasion may be associated with host proteins forming a surface coat, also known as antigenic disguise. Alderete and Baseman described that both rabbit and human albumins, as well as other host proteins, could be adsorbed on the surface of TPA (213). Egesten et al. showed that Group G streptococci express protein G on their surface, and it is able to bind human albumin, and therefore, inhibit anti-microbial action of CXCL9 (214). Accordingly, it has been proposed that the capacity of Tp0483 to bind soluble fibronectin could be related to the evasion of the humoral response (164). However, Cox and colleagues did not detect serum proteins on the surface of TPA by immunoelectron microscopy (26), stating that the low density of OMP might be the main immune evasion mechanism of TPA.

## 7 Discussion

Syphilis is a multistage disease caused by TPA, for which an effective vaccine remains elusive. Despite several decades of research, the impossibility to genetically manipulate and grow TPA *in vitro*, the paucity of OMPs, the OM fragility, and the lack of an appropriate animal model that recapitulates all stages of human syphilis have hampered the field progress. In addition, many controversies remain unresolved with respect to OMPs identification, location, function, the potential of these proteins to induce protective immune responses, or the characteristics of these responses. Researchers have hypothesized that a DTH immune response is essential to control and clear TPA infection, but it is also responsible for the development of tissue damage, including an ulcerative lesion that forms chancres. As DTH is primarily mediated by Th1 CD4^+^ T cells, the presentation of antigens by DCs, macrophages, or other professional antigen-presenting cells is crucial. Of note, this process is independent of antigen location, and both intracellular and surface-exposed antigens can be indistinctly processed and presented to T cells. Therefore, both antigens might generate protective Th1 CD4^+^ T cell responses. Thus, rabbits immunized with intracellular endoflagellar antigens that have been directly isolated from TPA showed faster development of lesions (215). However, bacteria were not detected in these lesions, which may be explained by an accelerated memory DTH response. In contrast, rabbits immunized with a plasmid coding for FlaB3 showed attenuated lesion development that was associated with an enhanced cellular infiltration, and an inhibition of bacteria dissemination to distal organs (216). In line with this observation, rabbits immunized with TprF, a periplasmic protein, displayed attenuated skin lesions with reduced bacteria burden (136). The reasons behind these different outcomes are not completely understood, but differences in vaccine formulation and delivery routes, the magnitude of vaccine-induced T cell responses, and the relative representation of antigens (flagellin and TprF) within the whole TPA proteome could be involved. Similar results to TprF immunization were also observed when OMPs were used as antigens. Therefore, DTH could be a protective immune response that modifies lesion development and clears bacteria from infection sites. However, none of TPA proteins tested as immunogens to date protected against infection, indicating that additional immune responses are likely to be required.

Despite some controversy (68), strong evidence supports a protective role of antibodies, facilitating bacteria opsonization and killing by innate immune cells, as well as blocking their interaction with components of the extracellular matrix, such as laminin and fibronectin. These functions strictly depend on the exposure of antigens on the OM and, in some cases (e.g. bacteria phagocytosis), the interaction with Fcγ receptors expressed on the surface of innate immune cells, such as macrophages. IgG-FcγR interaction may also improve antigen presentation by antigen-presenting cells and the generation of Th1 cellular responses (217). Therefore, to develop a protective TPA vaccine, it is crucial to define which types of antigens can induce synergic anti-TPA immune responses. It is also essential to considerer immune evasion strategies developed by TPA, such as functional redundancy and phase variation. In this sense, targeting several proteins with two or more functions, such as transport and adhesion, could increase vaccine efficacy, as it was previously observed (218, 219).

The ideal syphilis vaccine should provide protection against TPA infection. However, even if the vaccine is unable to completely prevent infection, it could still worth considering as a means of limiting bacteria dissemination and tissue invasiveness, blocking the establishment of latency, or preventing the disease from progressing to secondary and tertiary stages (113). Additionally, a vaccine that reduces bacterial persistence or prevents TPA transplacental invasion might also reduce the number of congenital syphilis in endemic areas, where the number of non-diagnosed infected individuals might be high, and the access to health care may be limited. Moreover, a syphilis vaccine has to be effective against possible reinfections with similar or different TPA strains or isolates, indicating the importance of using conserved antigens. Recently, there has been evidence that TPA can be cultured *in vitro* and that genetic manipulation of this pathogen can be accomplished (114, 220, 221). These scientific advances are likely to open the gateway to new research lines that can shed light on the current controversies.

## Author contributions

CÁ-N, OM, BC, JB and JC designed the scientific content. CÁ-N, NP-L and JC drafted the manuscript and figures. OM, BC and JB edited the manuscript and participate in content discussion. All authors have read and approved the content of the manuscript.
